# Comparing an Integrated Amphiphilic Surfactant to Traditional Hydrophilic Coatings for the Reduction of Catheter-Associated Urethral Microtrauma

**DOI:** 10.1021/acsomega.4c02109

**Published:** 2024-05-09

**Authors:** Jane Burns, David Pollard, Ased Ali, Colin P. McCoy, Louise Carson, Matthew P. Wylie

**Affiliations:** † School of Pharmacy, Queen’s University Belfast, 97 Lisburn Road, Belfast BT9 7BL, U.K.; ‡ Convatec Technology Centre, First Avenue, Deeside Industrial Park, Convatec Limited, Deeside, Flintshire CH5 2NU, U.K.

## Abstract

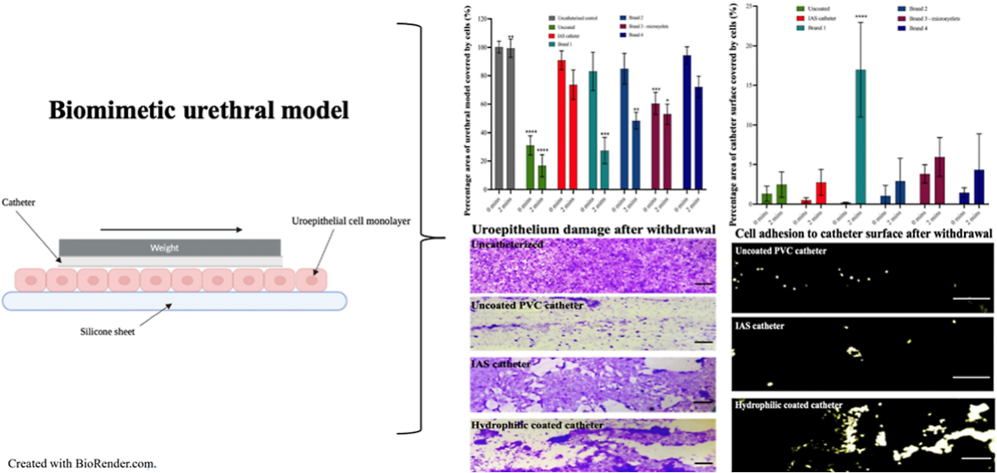

Hydrophilic-coated intermittent catheters have improved the experience of intermittent urinary catheterization for patients compared to conventional gel-lubricated uncoated catheters. However, the incorporation of polyvinylpyrrolidone (PVP) within hydrophilic coatings can lead to significant issues with coating dry-out. Consequently, increased force on catheter withdrawal may cause complications, including urethral microtrauma and pain. Standard methods of evaluating catheter lubricity lack physiological relevance and an understanding of the surface interaction with the urethra. The tribological performance and urethral interaction of commercially available hydrophilic PVP-coated catheters and a coating-free integrated amphiphilic surfactant (IAS) catheter were evaluated by using a biomimetic urethral model designed from a modified coefficient of friction (CoF) assay. T24 human urothelial cells were cultured on customized silicone sheets as an alternate countersurface for CoF testing. Hydrophilic PVP-coated and coating-free IAS catheters were hydrated and the CoF obtained immediately following hydration, or after 2 min, mimicking in vivo indwell time for urine drainage. The model was observed for urethral epithelial cell damage postcatheterization. The majority of hydrophilic PVP-coated catheters caused significantly greater removal of cells from the monolayer after 2 min indwell time, compared to the IAS catheter. Hydrophilic PVP-coated catheters were shown to cause more cell damage than the coating-free IAS catheter. A biomimetic urethral model provides a more physiologically relevant model for understanding the factors that govern the frictional interface between a catheter surface and urethral tissue. From these findings, the use of coating-free IAS catheters instead of hydrophilic PVP-coated catheters may help reduce urethral microtrauma experienced during catheter withdrawal from the bladder, which may lead to a lower risk of infection.

## 1 Introduction

Urinary retention is a serious disorder that is commonly associated with bladder outflow obstruction or neurogenic dysfunction, such as in patients with spinal cord injuries, traumatic brain injury, and multiple sclerosis.^1^ Clean intermittent self-catheterization is considered the gold standard treatment to relieve urinary retention^2^ and the use of this technique within Europe has doubled over the last two decades, largely due to a rise in continence care associated with an aging population, clinical benefits, and improved patient quality of life.^3,4^ Within the United Kingdom, intermittent catheters are typically single-use devices used by an estimated 60,000 people up to five times daily, requiring >57 million catheters annually.^5^

Intermittent catheters have historically been divided into two generations, differing in surface properties which may affect the risk of urethral complications such as patient discomfort, urethral microtrauma, hematuria, and an increased risk infection development.^6,7^ First-generation uncoated catheters require prelubrication with a sterile gel-based lubricant to aid insertion into the urethral opening, while the introduction of hydrophilic-coated intermittent catheters during the 1980s as a second-generation device aimed to improve the ease of catheter insertion with subsequent reduction in discomfort and trauma associated with catheterization.^8^ Hydrophilic-coated catheters are coated with hydrophilic polymers that allow rapid absorption of water to form a hydrated surface layer to reduce friction between the catheter surface and urethral tissue, and may also decrease the risk of catheter-associated urinary tract infection development, although the latter benefit is still inconclusive.^8,9^ Hydrophilic polymers such as poly(ethylene oxide)s or polyvinylpyrrolidones (PVPs) have been employed to achieve significantly reduced interfacial frictional forces compared to uncoated catheters but this benefit is usually short-lived due to the fragility of these coatings, with coating delamination commonly occurring during contact with urethral tissue.^10^ Moreover, PVP is the most commonly used polymer in hydrophilic coatings for intermittent catheters due to its ability to rapidly form a surface hydration layer upon contact with an aqueous-based hydrating solution. However, the lubricity of PVP is strongly linked to being fully wetted, with loss of hydration level to <75% weight water leading to promotion of mucoadhesive or sticky properties.^11^ This increased stickiness of the PVP-based catheters can lead to an increased withdrawal force to remove the catheter, increasing the potential for discomfort and urethral trauma. Previous literature investigating PVP-coated intermittent catheter use have reported a sticking sensation during catheter withdrawal, which can be linked to drying out of the PVP coating during catheterization.^12−15^ This can be particularly problematic for patients with limited hand mobility who may require up to 20 min to self-catheterize successfully^16^ and are therefore more likely to experience sticking during withdrawal of PVP-coated catheters.^2^

Recently, we described a novel coating-free alternative to traditional PVP-based catheters that possesses an integrated amphiphilic surfactant (IAS) technology in which the amphiphilic surfactant orientates its hydrophilic headgroup on the surface of the catheter to create a hydrophilic surface.^17^ Upon contact with an aqueous solution, the IAS technology can promote hydrogen bonding with the catheter surface to generate a hydration layer which provides lubrication for the duration of catheterization, avoiding the dry-out issues commonly associated with PVP-based coated catheters, such as coating dry-out, adhesion, and delamination.^18^

Humphreys et al. (2020) previously discussed the need to move beyond the current overreliance on solely mechanical assessments, such as ISO 8295:1995,^19^ to evaluate catheter surface lubricity and durability toward conditions which are more physiologically relevant and allow improved understanding of the factors that govern the frictional interface between a catheter surface and urethral tissue.^20^ This was emphasized by their inability to identify a link between the frictional performance of four hydrophilic coated catheters and urethral microtrauma when using their biomimetic model, which involved the use of a cell layer of human urethral epithelial cells in place of the traditional rubber countersurface employed in ISO 8295:1995.

In this current study, we aim to build on these findings by evaluating the impact of catheter dry-out and comparing the frictional performance of traditional PVP-coated catheters with an IAS catheter using a biomimetic urethral model. Humphreys et al. (2020) examined the intermittent catheters in their model after 30 s hydration in deionized water, whereas we also aim to evaluate the effect of residence time on catheter surface dry-out and microtrauma.^20^ Moreover, we will exploit this model to allow detailed assessment of catheter surfaces postcatheterization to determine if the contrasting hydrophilic surface technologies result in differences in both urethral epithelial cell damage as well as the adhesion of cells to the catheter surface to elucidate further understanding of frictional interface between these surfaces.

## 2 Materials and Methods

### 2.1 Materials

Six male intermittent catheters of CH12 size were evaluated: four hydrophilic PVP-coated catheters (Coloplast SpeediCath Flex, Coloplast Luja, Hollister VaPro Pocket and Wellspect LoFric Origo), an IAS catheter (GentleCath Glide with FeelClean Technology-Convatec Ltd., Deeside, UK), and an uncoated catheter (Self-Cath-Coloplast Ltd., Peterborough, UK). T24 bladder carcinoma cells [LCL-1709, American Type Culture Collection (ATCC) HTB-4] were purchased from ATCC (Virginia, US). McCoy’s medium, penicillin–streptomycin (10,000 U/mL), Trypsin-ethylenediaminetetraacetic acid (0.5%), 10% fetal bovine serum (FBS), toluidine blue-O (>80%), phosphate-buffered saline (PBS), paraformaldehyde (>99%), and fibronectin (>95%) were obtained from Sigma-Aldrich (Ireland Limited, Wicklow, Ireland). Hoechst stain (2 μg/mL 33258, bisbenzimide) was purchased from Abcam (Cambridge, UK). Red food coloring was bought from a high-street store.

### 2.2 Biomimetic Model

#### 2.2.1 Cell Seeding

T24 bladder carcinoma cells (LCL-1709, ATCC HTB-4) were maintained in complete growth media, McCoy’s 5A medium supplemented with 10% FBS and 50 U/mL pen strep. Cells were cultured under standard tissue culture conditions of 37 °C and 5% CO_2_ atmosphere. Cells were not passed above passage number 18 to prevent genetic drift.^21^

Silicone sheets (approximately 7 × 10 × 0.2 cm surrounded by a 0.5-cm high and 1-cm thick perimeter wall) were customized as a countersurface base. Elastosil LSR-4350 silicone elastomer parts A and B were mixed at a ratio 1:1 and manipulated between glass plates for curing at 90 °C overnight. The silicone sheets were sterilized by autoclaving before being placed into square 15 × 15 cm cell culture Petri dishes (Corning, Arizona, US). To increase the surface hydrophilicity, the silicone sheets were coated with 4 μg/cm^2^ fibronectin in deionized water for 24 h. The fibronectin was rinsed with PBS and T24 cells were seeded on to the customized silicone sheets 50 × 10^3^ cells per cm^2^.

#### 2.2.2 Coefficient of Friction Analysis

**Figure 1:**
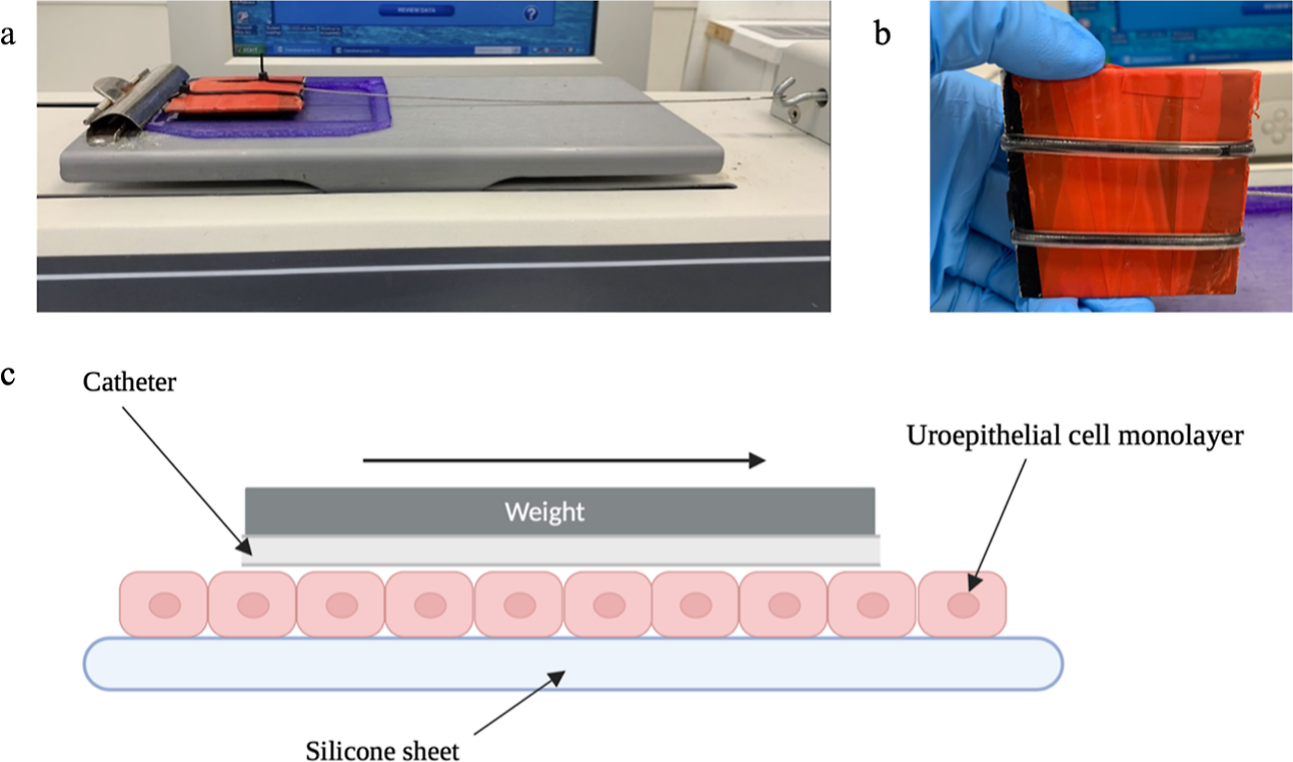
(a) Image of biomimetic model setup. (b) Image of catheter segments attached to weight. (c) Diagram illustrating the biomimetic model created with BioRender.com.

A modified coefficient of friction (CoF) assay was adapted from ISO 8295:1995 and used to assess urethral microtrauma resulting from the use of intermittent catheters. This was achieved using T24 cell monolayers cultured on the customized silicone sheets as a counter surface on the platform of a CoF apparatus (Model COF-1000, ChemInstruments, Ohio, US) (Figure 1).^19^ After hydrating the catheters as per the manufacturer guidelines, the noneyelet portion of the catheters was cut longitudinally into 6 cm segments and attached to a 15 g weight using cable ties. In practice, uncoated catheters are usually gel lubricated manually; however, herein, the catheter was left unlubricated to act as a negative control. Two segments were attached at a time for weight stability (Figure 1). Noneyelet sections of the catheters were tested. Catheters advanced across the cell monolayer at 15 cm min^–1^ for 5 cm immediately after hydration and attachment (0 min). To mimic varying indwelling times and potential of catheter surface dry-out, catheters were also left in contact with the T24 cell monolayer for 2 min before advancement at 15 cm min^–1^ for 5 cm. The resulting data was analyzed accordingly to determine both static and kinetic CoF.^20,22^

#### 2.2.3 Analysis of Uroepithelium Damage

Cell monolayers were visually examined for detachment and loss of confluency by staining and light microscopy. Cell staining with 0.1% v/v crystal violet solution was performed after the urethral model was catheterized by each commercial catheter for a visual examination of detachment and loss of confluency. A Leica Digital Microscope-GES (Leica, Wetzlar, Germany) was used to image the “catheterization” tracks across the cell seeded silicone substrate. The total surface area of the cells remaining on the silicone substrate after “catheterization” was quantified using ImageJ software, Measure function, and displayed as the percentage (%) area covered by cells. Results were normalized to the catheterized control group, allowing for threshold processing in ImageJ.

##### 2.2.3.1 Blinded Visual Scoring of Cell Damage after Catheterization

After catheterization of the cell monolayers by each catheter in the biomimetic model, cells on the “catheterization tracts” were imaged at ×40 magnification for closer visual examination of cell health. Cells were scored according to the cell detachment from the urethral model. A template for grading cell health was adapted from the Morphology grading of cytotoxicity in the ISO 10993-5 (biological evaluation of medical devices: tests for in vitro cytotoxicity; Table 1-qualitative morphological grading of cytotoxicity of extracts) (Table 1).^23^

**Table 1:** Qualitative Morphology Grading Scale

grading		condition of cells
0	none	cells remain adhered to substrate/in a cell monolayer
1	slight	<20% removal of cells from monolayer
2	mild	<50% removal of cells from monolayer
3	moderate	<70% removal of cells from monolayer, cell lysis or debris observed
4	severe	complete destruction and removal of cells from monolayer

To ensure the scoring of the cells after catheterization remained unbiased, a blinded visual study was conducted with 25 PhD students from the School of Pharmacy, Queen’s University Belfast. Using the grading scale (Table 1), the students were provided with blinded images of cells remaining on the monolayer post catheterization with each commercial intermittent catheter after 0 or 2 min precontact (*n* = 12). All students had a familiarity with microscopy and cell identification but had no previous knowledge of the current project or context of the study.

#### 2.2.4 Cell Adhesion to Catheter Surface

The attachment of urethral cells to each catheter surface was also examined by staining. Following catheterization of the urethral model, catheter samples were removed and fixed by adding 1 mL of 4% paraformaldehyde per sample for 20 min. Samples were rinsed with PBS and 1 mL of fluorescent Hoechst stain (2 μg/mL) was added. Cell attachment was then visually examined using a fluorescence microscope (GXM-L3201 LED, GX Optical, Suffolk, UK) with a ×40 objective, and images were taken. ImageJ software was used for quantification of the percentage surface area (%) covered by adherent cells. Cell counting was not performed due to areas of cell clumping, which made individual cells difficult to define. Areas corresponding to the attached cells (colored blue) were individually calculated and displayed as the percentage (%) of area covered by cells.

#### 2.2.5 Visual Assessment of Catheter Hydrophilic Coating Delamination

The catheters were stained with a 1:1 dye solution of deionized water and water-based red food coloring. A dowel was placed inside each catheter to prevent the uptake of dye into the catheter lumen. Each catheter segment was placed in the dye for 2 min and then immersed briefly in deionized water to remove any excess dye. After “catheterization” of cell monolayers in the biomimetic model, images were taken of the catheterization tracts and catheter surface for evidence of delamination.

### 2.3 Statistical Analysis

Statistical analysis was performed using Graph Pad Prism 9.0 for Mac (GraphPad Software Inc., San Diego, USA). Statistical differences between the static and kinetic CoF of each catheter on T24 cell monolayers, the percentage (%) area covered by cells postcatheterization, and the percentage (%) area covered by cells postcatheter contact was evaluated by a two-way analysis of variance test (ANOVA). Posthoc comparisons were performed using Tukey’s multiple comparisons test. In all cases, differences were considered significant when *p* < 0.05 (*n* = 12).

## 3 Results

### 3.1 In Vitro Assessment of Catheter Surface Lubricity

**Figure 2:**
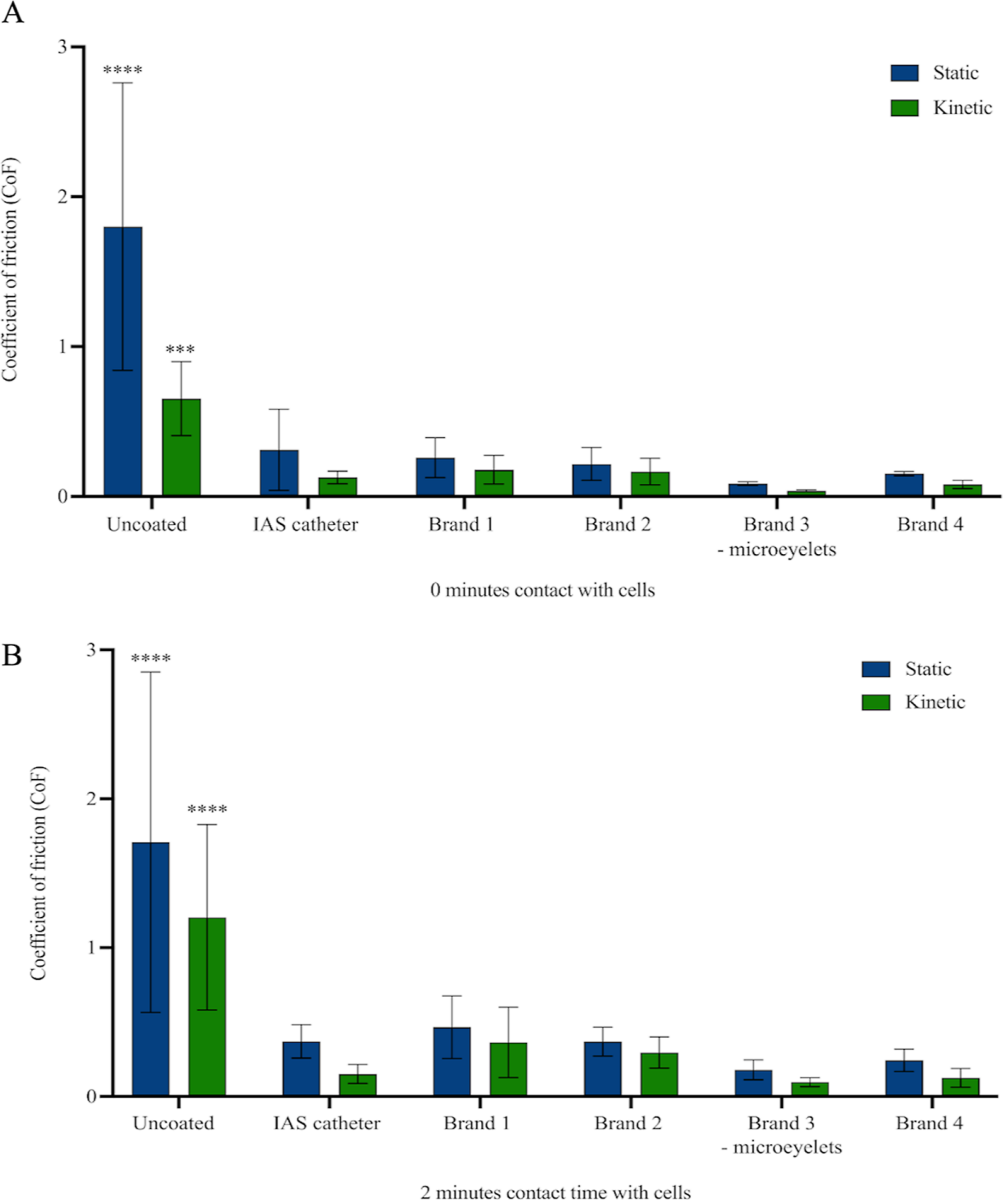
Mean static and kinetic CoF values (*n* = 12) determined for commercial intermittent catheters on a T24 cell-seeded silicone urethral model as a countersurface. Values were determined immediately after catheters were placed onto the cell countersurface at 0 min (A), to mimic catheter insertion, or after catheters were placed onto the cell countersurface for 2 min (B), to mimic catheter withdrawal. Error bars represent standard deviations. Statistical significance relative to the IAS catheter is indicated as *p ≤ 0.05; **p ≤ 0.01; ***p ≤ 0.001; and ****p ≤ 0.0001.

To assess the tribological performance, segments of intermittent catheters were fixed to a 15 g weight and advanced across T24 cell monolayers at 15 cm min^–1^ for a 5 cm CoF apparatus. Catheters were displaced across the monolayer surface at a controlled speed mimicking catheter insertion (0 min) and withdrawal (2 min) to determine the CoF, displayed in Table 2 and Figure 2.

**Table 2:** Mean Static and Kinetic CoF Values (*n* = 12) Determined for Commercial Intermittent Catheters on a T24 Cell-Seeded Silicone Urethral Model as a Countersurface^a^

	0 min		2 min	
	static CoF	kinetic CoF	static CoF	kinetic CoF
uncoated PVC catheter	1.80 ± 0.96	0.65 ± 0.25	1.71 ± 1.14	1.20 ± 0.62
IAS catheter	0.31 ± 0.27	0.13 ± 0.04	0.37 ± 0.11	0.15 ± 0.06
Brand 1	0.26 ± 0.13	0.18 ± 0.10	0.47 ± 0.21	0.36 ± 0.24
Brand 2	0.22 ± 0.11	0.17 ± 0.09	0.37 ± 0.10	0.30 ± 0.10
Brand 3-micro eyelets	0.09 ± 0.01	0.04 ± 0.01	0.18 ± 0.07	0.10 ± 0.03
Brand 4	0.15 ± 0.01	0.08 ± 0.03	0.24 ± 0.07	0.13 ± 0.06

a Values were determined immediately after catheters were placed onto the cell countersurface at 0 min, or after catheters were placed onto the cell countersurface for 2 min, to mimic in vivo indwell time.

The CoF of the uncoated catheter was significantly greater after 0 and 2 min than all of the other hydrophilic catheters on the cell-seeded countersurface. Both kinetic and static CoF measurements of the uncoated catheter were at least threefold greater than the IAS and PVP-coated catheters, as expected, due to lack of surface lubricity (Figure 2). This reflected the findings observed by Humphreys et al. (2020), where upon hydration of the intermittent catheters and testing in their model, the uncoated catheter demonstrated the greatest CoF.^20^ No significant difference was found between the static and kinetic CoF of the IAS catheter and the hydrophilic PVP-coated catheters at either 0 or 2 min cell contact time. Despite this, Brand 3 (microeyelets) demonstrated the lowest CoF values throughout, indicating an increased lubricity. Whereas Brand 1 demonstrated the highest CoF values at 0- and 2 min cell contact time out of the hydrophilic catheters, except for the static CoF of the IAS catheter at 0 min (0.31 ± 0.27). Notably, the IAS catheter demonstrated hydrophilic properties comparable to those of the hydrophilic PVP-coated catheters, despite the lack of a hydrophilic coating. Overall, it was shown that the CoF of the IAS catheter and the hydrophilic PVP-coated catheters increased from 0 to 2 min cell contact time. This was predicted due to the expected catheter surface dry-out.

### 3.2 In Vitro Assessment of Microtrauma

**Figure 3:**
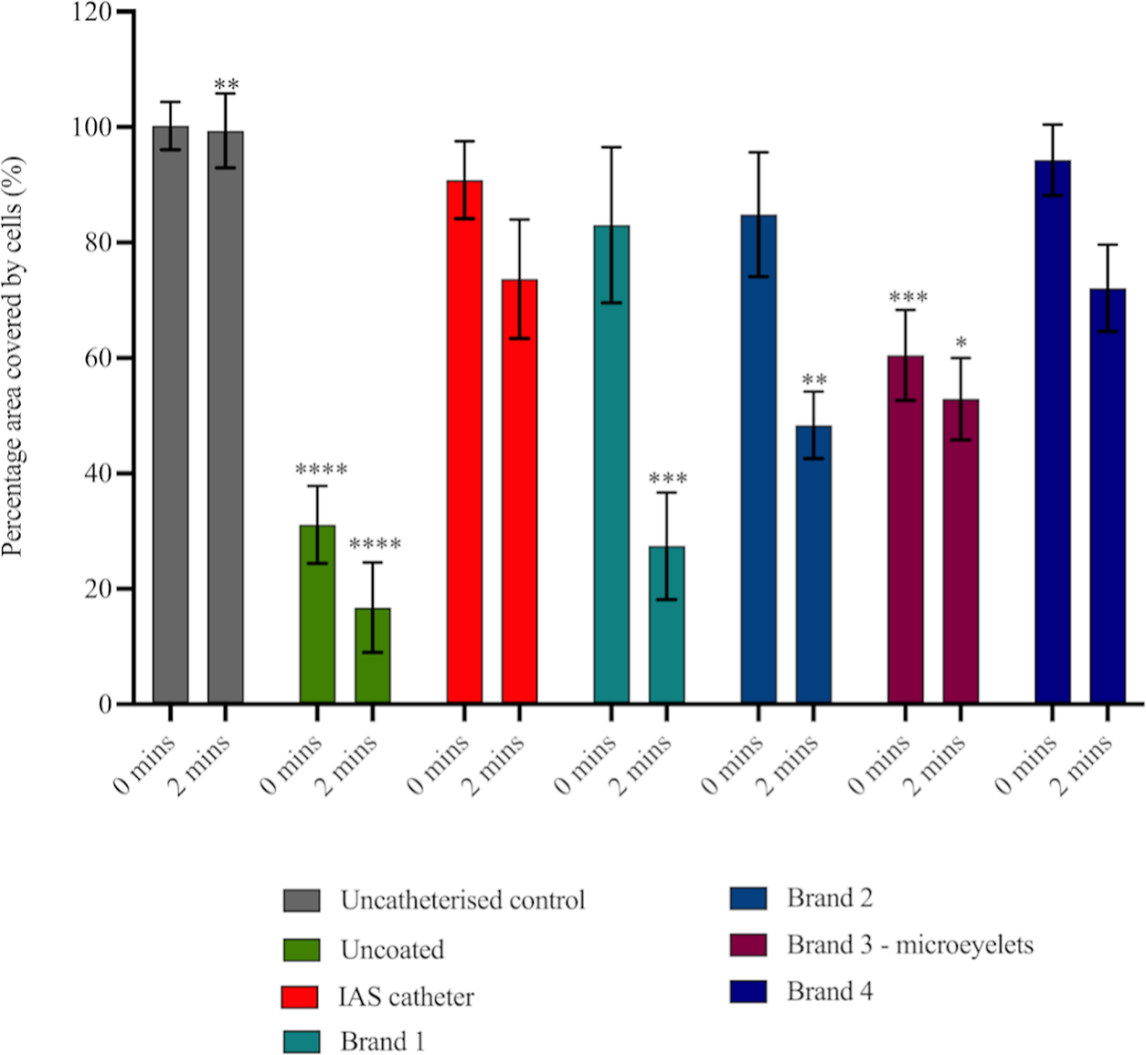
Percentage (%) area of the urethral model that was covered with T24 cells post catheterization, normalized to the uncatheterized control (*n* = 12). Images were taken post catheterization, and the surface area of cells remaining was calculated relative to the control. Error bars represent standard deviations. Statistical significance relative to the IAS catheter are indicated as *p ≤ 0.05; **p ≤ 0.01; ***p ≤ 0.001; and ****p ≤ 0.0001.

**Figure 4:**
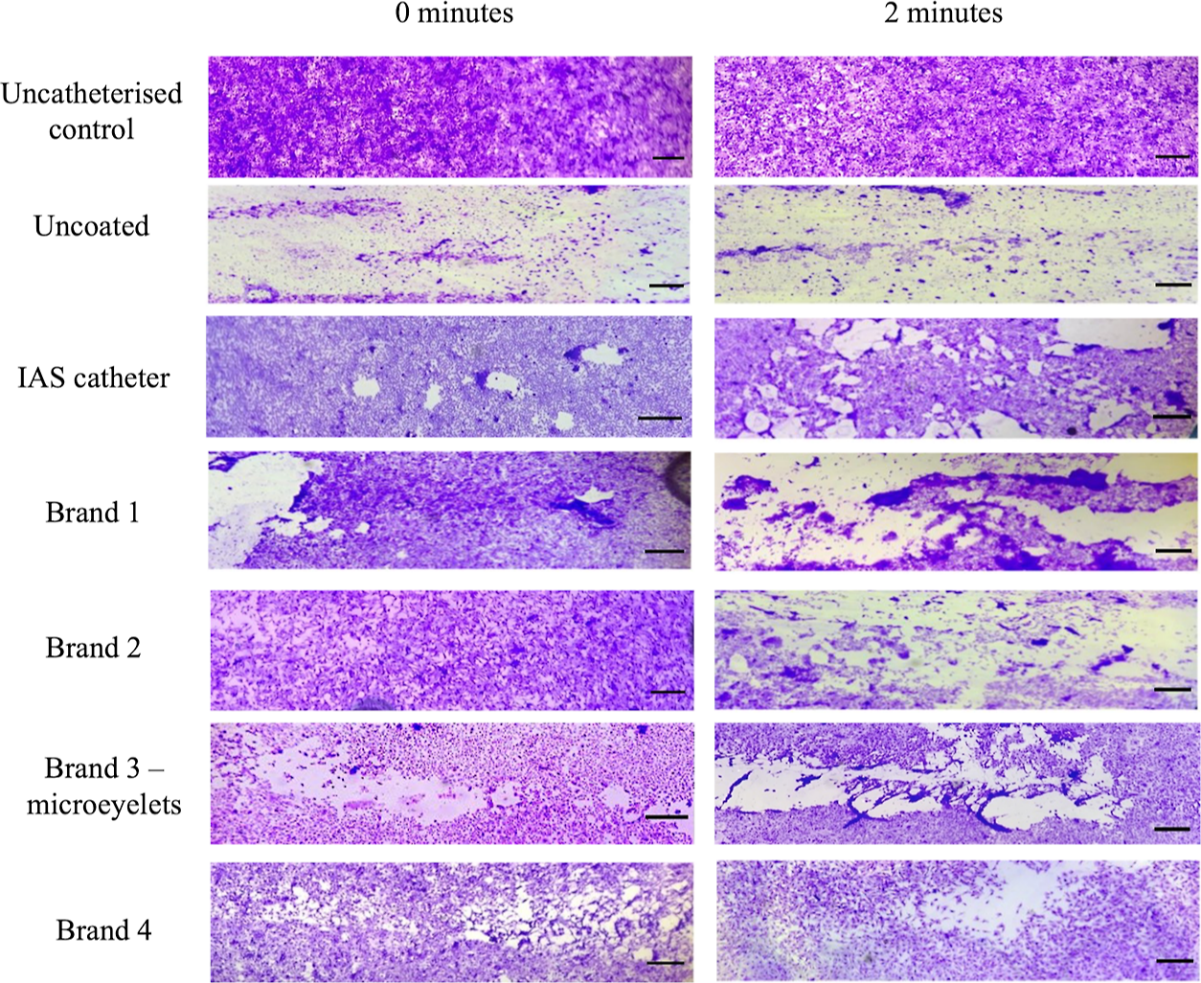
T24 monolayers were seeded on silicone substrates for CoF measurements. After catheterization in the urethral model, cells were stained with 0.1% v/v crystal violet solution. Images acquired with light microscopy at ×25 magnification. Control: T24 cell monolayers seeded on silicone substrates without catheterization. Scale bars 100 μm. Representative images from 12 replicates are shown.

To assess the effect of intermittent catheters on microtrauma, cell monolayers were visually examined postcatheterization for detachment and loss of confluency by staining and light microscopy. The total surface area of the cells remaining on the silicone substrate after “catheterization” (catheterization tracts) was quantified using ImageJ and the percentage (%) area covered by cells relative to uncatheterized control displayed in Figures 3 and 4.

Stained cells remaining on the urethral model post catheterization are shown in Figure 4. The uncoated catheter exerted the most damage to the cell monolayer as expected due to high friction between the uncoated PVC surface and cells. Damage to the cell monolayer with almost complete exposure of the silicone substrate was observed indicating that enough force to overcome cell adhesion was exerted. This correlates with the high CoF measurements recorded in Table 2.

After catheterization of the urethral model at 0 min (mimicking catheter insertion), no difference was found between the percentage of cells remaining postcatheterization with the IAS catheter, Brand 1, Brand 2 and Brand 4. Moreover, the mean percentage of cells remaining postcatheterization with the IAS catheter, Brand 1, Brand 2 and Brand 4 was similar to the uncatheterized control, indicating mild damage to the cell monolayer. The shear force exerted by the catheter surface was insufficient to fully overcome the adhesion strength of the cells, but it was great enough to cause removal of some cells, resulting in a lower total measured percentage.

The mean percentage of cells remaining on the urethral model postcatheterization after 2 min, mimicking in vivo indwell time, with all the commercial catheters significantly decreased, compared to the uncatheterized control. All catheters exerted damage to the cell monolayer indicating enough physical force was employed to disrupt cell adhesion to the silicone substrate. However, despite this the IAS catheter caused significantly less removal of cells from the urethral model and, therefore less cell damage, than the uncoated, Brand 1, Brand 2 and Brand 3 (microeyelets) catheters.

Interesting observations at each cell contact time in the urethral model were that first, Brand 3 (microeyelets) was the only PVP-coated catheter to cause significantly more damage to the cells than the IAS catheter after 0 min catheterization. Second, after 2 min of catheterization, Brand 4 caused significantly less cell damage than the other PVP-coated catheters, performing similar to the IAS catheter. However, both Brand 3 (microeyelets) and Brand 4 demonstrated the lowest CoF values throughout assessment of catheter lubricity (Table 2). This indicates that the cause of urethral microtrauma may not be solely a consequence of intermittent catheter surface lubricity. Similar observations were previously stated. Humphreys et al. (2020) found that after testing four hydrophilic coated intermittent catheters in their biomimetic model, despite the CoF of Brand C being twice that of Brand A and B, no increase in damage to the cells in the model was observed. Humphreys et al. suggested that the relationship between urethral irritation and microtrauma may be intricate and not simply attributed to surface lubricity and our findings within this study reinforce this conclusion.^20^

In general, cell removal from the monolayer increased from 0 to 2 min cell contact time. Again, this may be due to the dry-out of the catheters’ surface. The increase in the contact time may be sufficient time for the catheter surfaces to begin to dry out, losing lubricious properties and resulting in damage to the cell monolayer (Figure 4). In particular, the hydrophilic PVP-coated catheter surface may dry out enough for the coating to become adhesive, causing increased friction between the catheter surface and cell monolayer.

#### 3.2.1 Blinded Visual Scoring of Cell Damage after Catheterization

**Figure 5:**
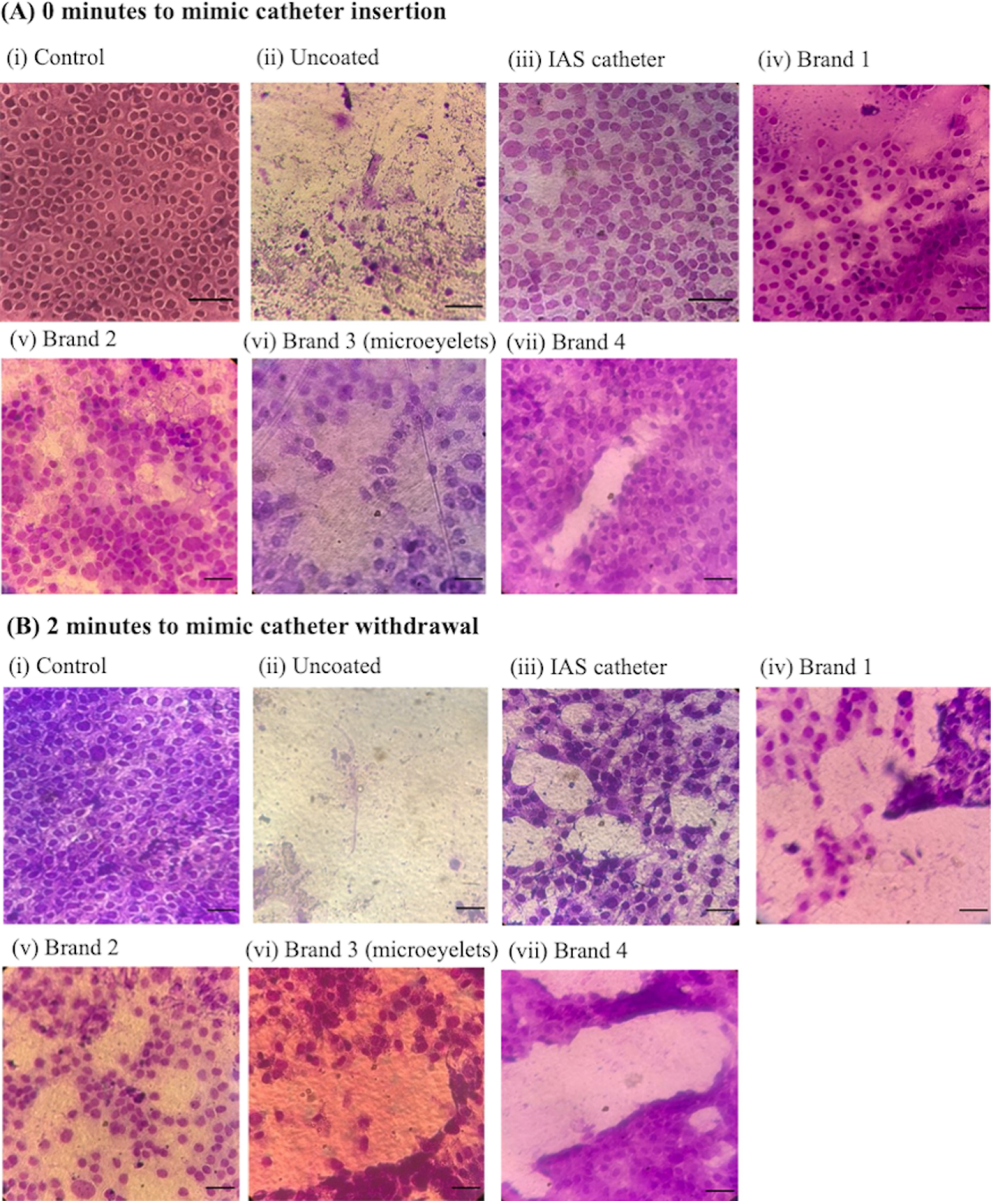
T24 cell damage postcatheterization: cells remaining on the urethral model postcatheterization. Catheters were placed onto the cell countersurface and advanced at 0 min (A), to mimic catheter insertion, or placed onto the cell countersurface for 2 min (B) and advanced, to mimic catheter withdrawal. Cells were stained with 0.1% v/v crystal violet solution. (i) Uncatheterized control, (ii) uncoated, (iii) IAS catheter, (iv) Brand 1, (v) Brand 2, (vi) Brand 3 (microeyelets) and, (vii) Brand 4. Images were acquired at ×40 magnification. Scale bar 10 μm. Representative images from 12 replicates are shown.

To assess the extent of cell damage exerted by intermittent catheters during catheterization, cell monolayers were visually examined postcatheterization in the biomimetic model. Cells were scored according to cell detachment from the urethral model by using a qualitative morphology grading scale (Table 1). Scoring was conducted by 25 PhD students from the School of Pharmacy, Queen’s University Belfast, in a blinded visual study, Table 3. Example images from blinded study are shown in the Supporting Information, Figure 1. Representative images are displayed in Figure 5.

**Table 3:** Qualitative Morphology Grading of T24 Cells Remaining on the Silicone Urethral Model Countersurface after Catheterization by Commercial Intermittent Catheters^a^

	qualitative morphology grading	
	0 min	2 min
uncoated catheter	4	4
IAS catheter	1	2
Brand 1	2	3
Brand 2	2	3
Brand 3 (microeyelets)	2	3
Brand 4	2	3

a Catheters were placed onto the cell countersurface and advanced at 0 min, to mimic catheter insertion, or placed onto the cell countersurface for 2 min and advanced, to mimic catheter withdrawal.

After catheterization of the cell monolayers by each catheter, cells remaining on the urethral model “catheterization tracts” were imaged stained with 0.1% v/v crystal violet solution and imaged at ×40 magnification for closer visual examination of cell health. In order to provide a nonbiased scoring of damage exerted to the cells by each catheter, 25 PhD students were provided with blinded images of cells remaining on the monolayer post catheterization and a grading scale (Table 1). Cells were scored according to detachment from the urethral model. Grading scored ranged from 0—None; cells remain adhered to substrate in a cell monolayer, to 4—Severe; complete destruction and removal of cells from the monolayer.

At both 0 min (catheter insertion) and 2 min (catheter withdrawal; mimicking in vivo indwell time), damage exerted to the cell monolayer in the urethral model by the uncoated catheter was graded 4, indicating severe and complete destruction. This was similar to previous observations of the catheterization tracts in Figure 4. Interestingly, volunteers in the study graded the IAS catheter to exert slight to mild damage, whereas all the hydrophilic PVP-coated catheters were graded mild to moderate. Although the IAS catheter was indicated to cause less cell damage than the hydrophilic PVP-coated catheters, all intermittent catheters were noted to cause cell damage to some extent, nonetheless.

### 3.3 Cell Adhesion to Catheter Surface

**Figure 6:**
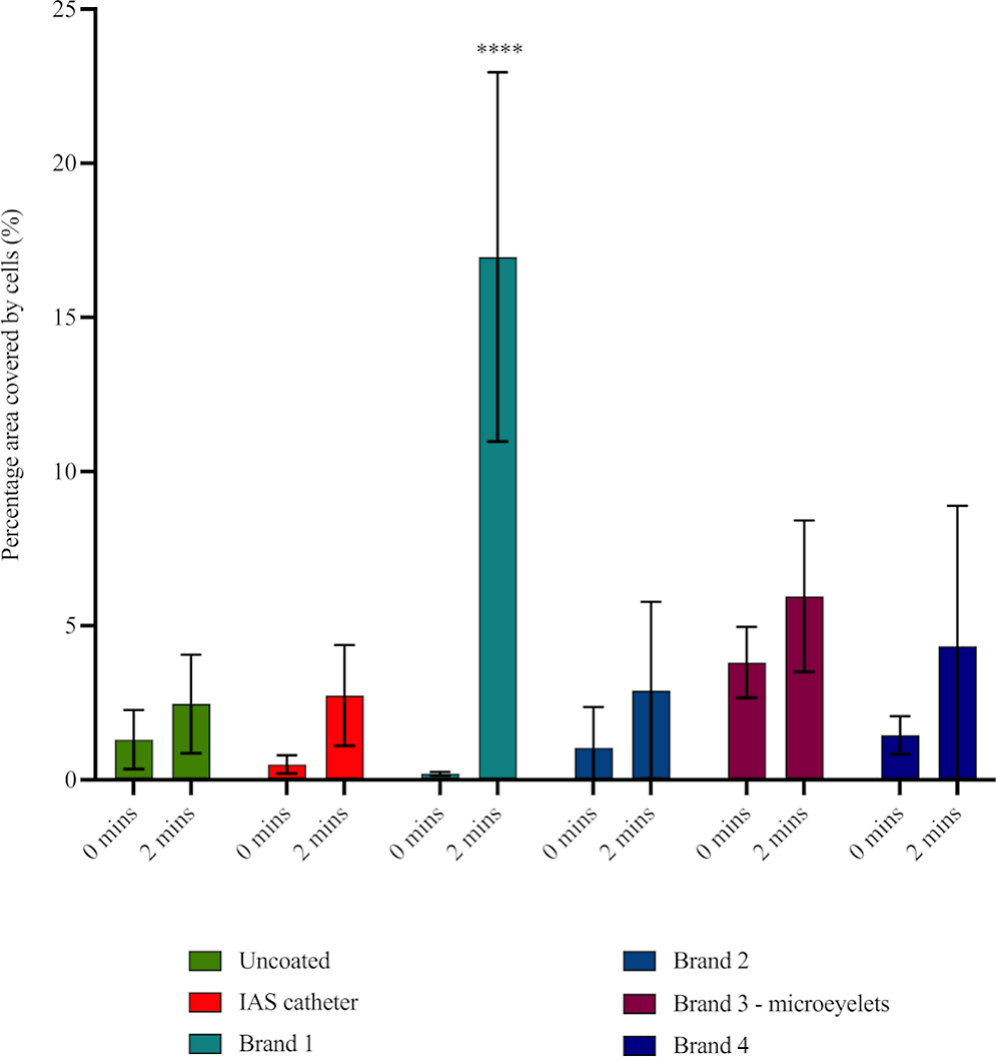
Percentage (%) area of the catheter surface that was covered with adhered T24 cells postcatheterization, (*n* = 12). Catheters were placed onto the cell countersurface and advanced at 0 min, to mimic catheter insertion, or placed onto the cell countersurface for 2 min and advanced, to mimic catheter withdrawal. Images were taken postcatheterization, and the surface area of cells remaining calculated. Error bars represent standard deviations. Statistical significance relative to the IAS catheter are indicated as *p ≤ 0.05; **p ≤ 0.01; ***p ≤ 0.001; and ****p ≤ 0.0001.

**Figure 7:**
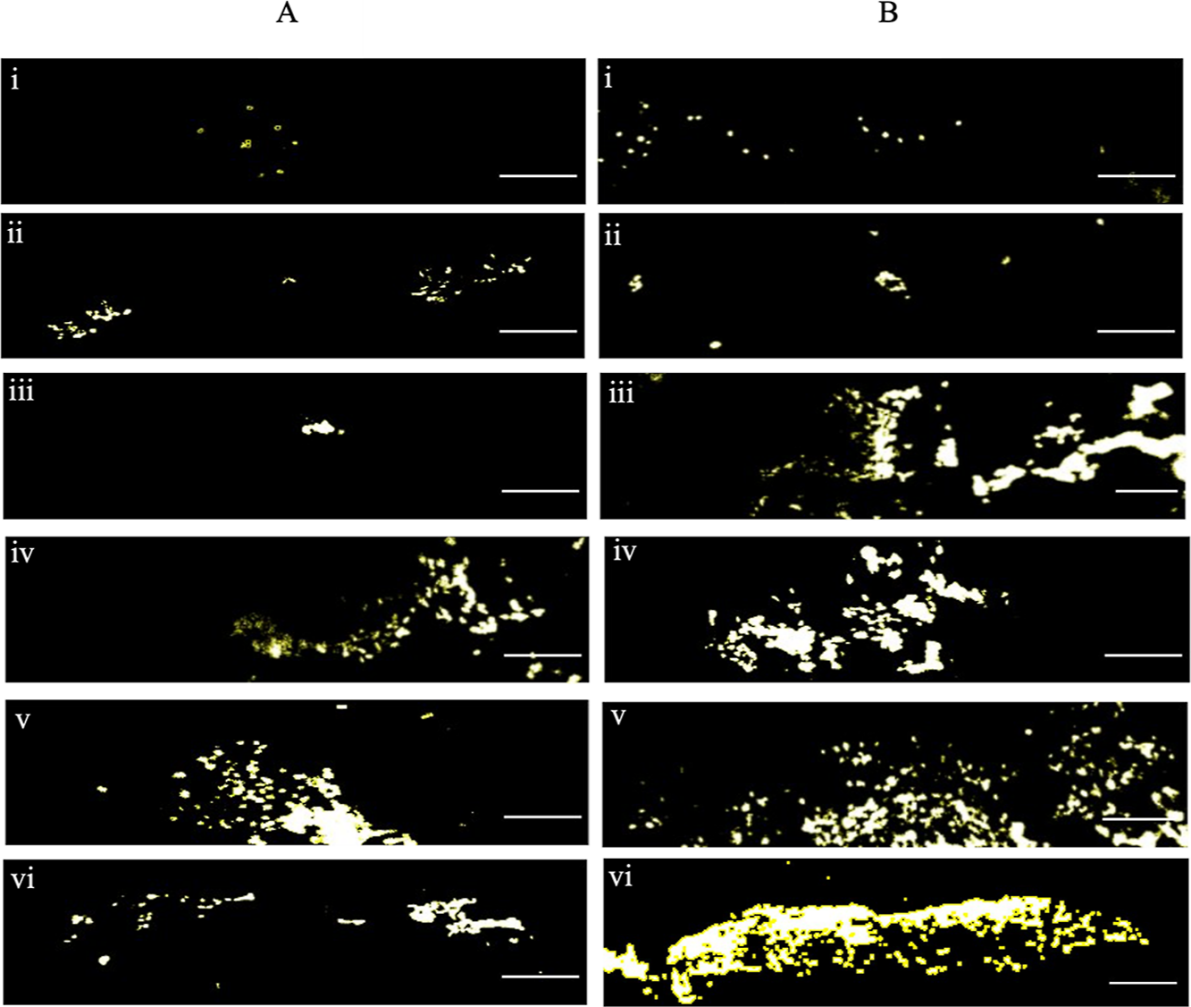
Cells attached to catheter surface post catheterization. Catheter segments were pulled horizontally at a speed of 15 cm/min for 5 cm over T24 cell monolayers and removed after (A) 0 and (B) 2 min. Cells were stained with 0.2% Hoechst stain solution and shown in ImageJ software; grayscale, create selection. (i) Uncatheterized control, (ii) uncoated, (iii) IAS catheter, (iv) Brand 1, (v) Brand 2, (vi) Brand 3 (microeyelets), and (vii) Brand 4. Images acquired at ×40 magnification. Scale bar = 100 μm. Representative images from 12 replicates are shown.

The attachment of urethral cells to each catheter surface was also examined by fluorescent staining. Following catheterization of the urethral model, catheter samples were removed and stained with a fluorescent Hoechst stain. Cell attachment was then visually examined using a fluorescence microscope, and images were taken. ImageJ software was used for quantification of the percentage surface area (%) covered by adherent cells (Figures 6 and 7).

The attachment of urethral cells to each catheter surface was also examined by Hoechst staining. Areas corresponding to the attached cells were displayed (Figure 7) and calculated as the percentage (%) area covered by cells (Figure 6). The uncoated catheter did not show a high percentage of cell coverage adhered to the surface. This was thought to be due to the lack of coating and, therefore, does not exhibit coating dry-out or adhesion. The friction exerted on the uncoated surface is enough to remove cells from the urethral model, but it does not possess an adhesive coating for the cells to stick to.

No significant difference was found between cell adhesion to the IAS catheter and the other hydrophilic PVP-coated catheters (Brand 2, Brand 3 [microeyelets] and Brand 4) at 0 or 2 min. However, the extent of cell adhesion to the catheter surface of Brand 1 was significantly greater (*p* ≤ 0.0001) postcatheterization at 2 min, mimicking in vivo indwell time. This was unexpected as it was thought that the hydrophilic PVP-coated catheters would perform similarly, with greater cell adhesion over time due to an adhesive surface. Loss of water from hydrophilic coated catheter is known to cause “sticky” surfaces.^24^

Previously, Brand 1, Brand 2, and Brand 3 (microeyelets) caused significantly greater damage to the cell monolayer after postcatheterization at 2 min (catheter withdrawal) (Figure 3) and notable removal of cells from the cell monolayer (Figure 4). Yet, as stated, only Brand 1 demonstrated greater cell adhesion to the catheter surface postcatheterization at 2 min, mimicking catheter withdrawal (Figure 6).

### 3.4 Visual Assessment of Catheter Hydrophilic-Coating Delamination

**Figure 8:**
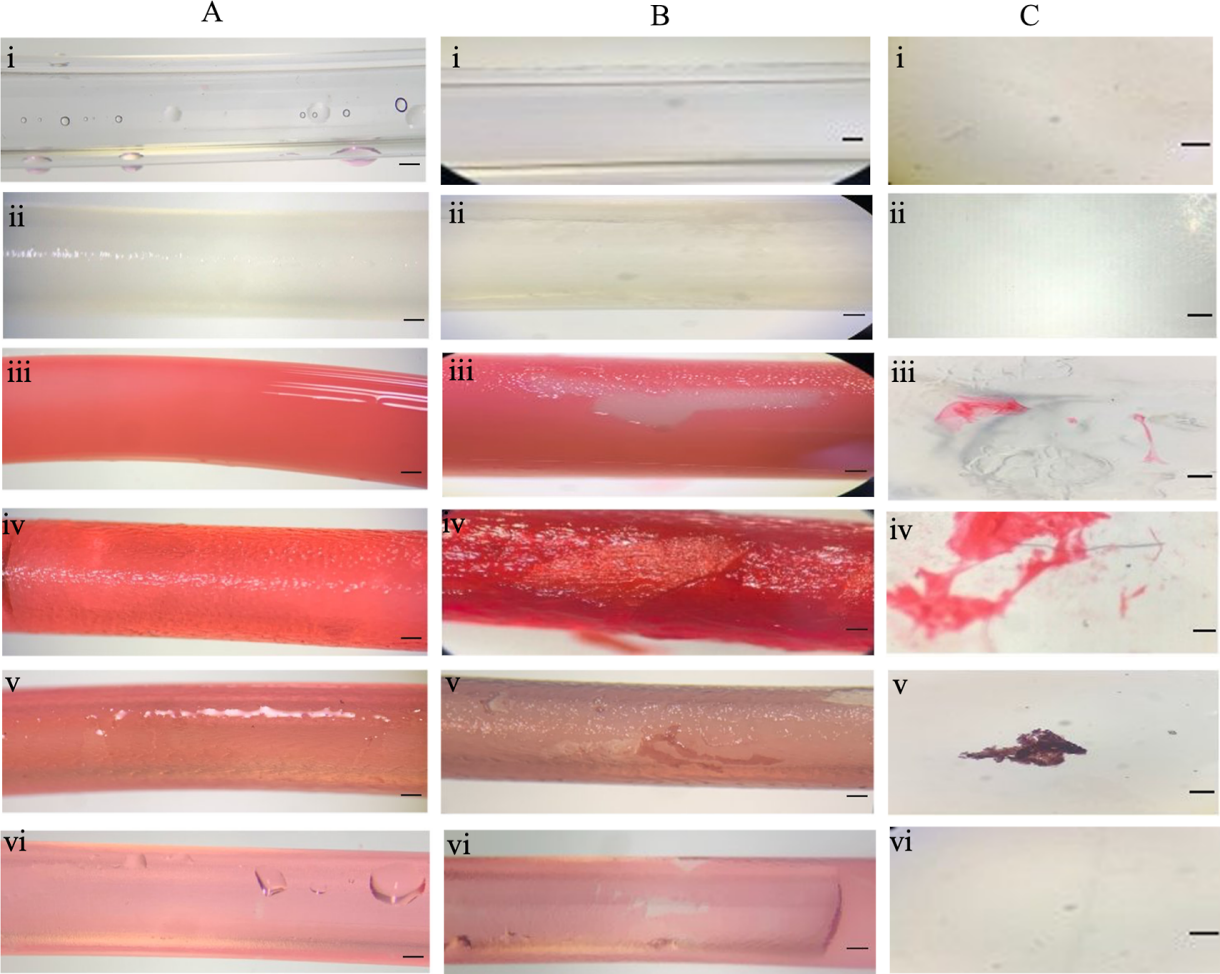
Images of (A) catheter surfaces immediately hydration and before catheterization, 10× magnification. (B) Catheter surfaces after 2 min catheterization, 10× magnification. (C) Coating residue on the cell-seeded silicone urethral model after catheterization using light microscopy, 30× magnification. (i) Uncoated, (ii) IAS catheter, (iii) Brand 1, (iv) Brand 2 (v) Brand 3 (microeyelets), and (vi) Brand 4. Scale bar (A) 1 mm, (B) 1 mm, and (C) 1 mm. Representative images from 12 replicates are shown.

To examine hydrophilic-coating delamination, catheters were stained with a water-based red food coloring and the surface imaged before and after “catheterization” of cell monolayers in the biomimetic model. Images were taken of the catheterization tracts and catheter surface for evidence of delamination and are shown in Figure 8.

**Figure 9:**
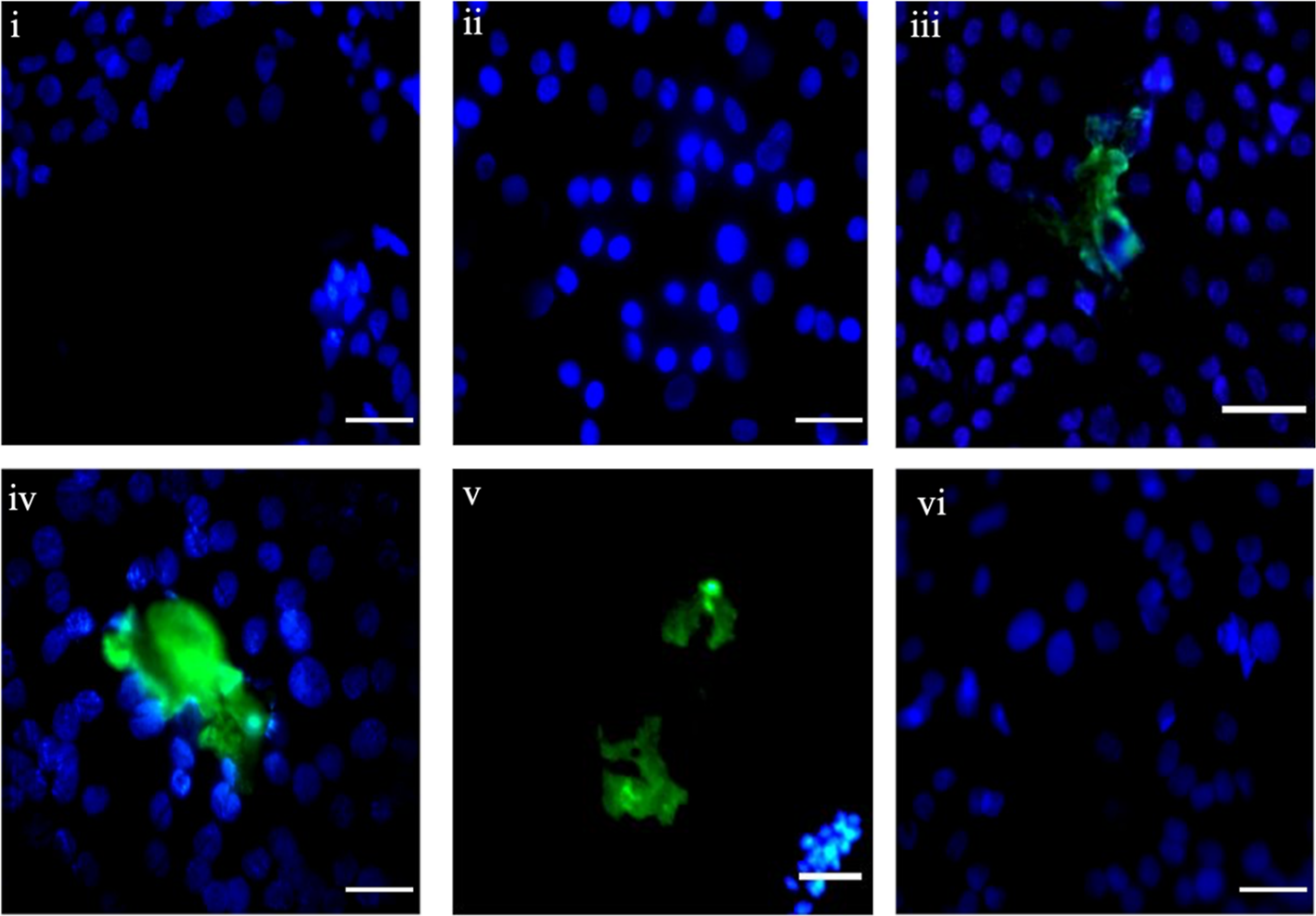
Coating residue on the cell-seeded silicone urethral model after 2 min catheterization. 0.2% Hoechst stain solution was added to the urethral model, and images were taken using fluorescence microscopy. Areas of coating residue appear green, T24 cells stain blue. (i) Uncoated, (ii) IAS catheter, (iii) Brand 1, (iv) Brand 2 (v) Brand 3 (microeyelets), and (vi) Brand 4. Images acquired at ×40 magnification, scale bar 10 μm. Representative images from 12 replicates are shown.

Fluorescent Hoechst stain was added to the catheterization tracts after catheterization to visualize the interaction between urethral cells and hydrophilic coating delamination in the biomimetic model. Images were taken and are shown in Figure 9.

Postcatheterization, all the hydrophilic PVP-coated catheters showed visible signs of coating delaminated from the catheter (Figure 8B). Additionally, after catheterization with Brand 1, Brand 2 and Brand 3 (microeyelets), catheter coating residue remained behind on the urethral model surface (Figure 8C). No coating delamination was observed on the uncoated catheter and the IAS catheter due to the lack of hydrophilic coating. Interestingly, Brand 4 did not appear to leave coating residue behind on the urethral model despite the catheter having a hydrophilic PVP coating and showing areas of coating delamination [Figure 8B(vi)]. The coating may begin to peel from the catheter surface but clump and remain adhered to the adhesive catheter shaft as it is removed from the model. Variables that may cause the difference seen in coating delamination between brands of hydrophilic PVP-coated catheters include the amount and type of PVP incorporated within the coatings and hydration solutions used. For example, Lundgren et al. (2000) reported a difference in frictional performance when catheters were hydrated with a saline solution rather than water.^25^

Fluorescent Hoechst staining was then used to observe the interaction between T24 cells remaining on the model and the coating residues left behind after catheterization. Using fluorescent microscopy, cells appeared blue, and the red dyed PVP-coating appeared green (Figure 9). Images mirrored those in Figure 8C showing the catheter coating residue of Brand 1, Brand 2, and Brand 3 (microeyelets), remaining behind on the urethral model surface after catheterization. Similarly, no coating residue was observed on the model after catheterization with the uncoated IAS and Brand 4 catheters. Interestingly, the PVP coating residues of Brand 1 and Brand 2 can be seen among the cells remaining on the model whereas Brand 3 (microeyelets) shows coating residue in areas void of cells, indicating they have been removed from the monolayer during catheterization. This may help explain why cell adherence to hydrophilic PVP-coated catheters Brand 2, Brand 3 (microeyelets) and Brand 4 was not significantly different to the IAS catheter (Figure 6). The coating, with cells embedded, sloughs off and remains behind the urethral model after catheterization.

## 4 Discussion

The aim of this paper was to evaluate and compare the tribological performance and urothelial interaction of traditional hydrophilic PVP-coated catheters with an IAS catheter using a biomimetic urethral model. Moreover, we wanted to assess the catheter surfaces postcatheterization, to determine if the contrasting hydrophilic and amphiphilic surface technologies result in differences in adhesion of cells to the catheter surface for a better understanding of the frictional interface and relationship between these surfaces. The extent of research into catheter-associated urethral microtrauma is limited.^18,20^ The CoF assay, while a widely accepted standard for assessing the lubricity of intermittent catheters, lacks the ability to simulate moist urethral conditions or potential urethral microtrauma. In this study, our model enables a more relevant in vitro assessment and comparison of commercial intermittent catheters in terms of their lubricity and impact on urethral microtrauma. Similar to Humphreys et al. (2020), we demonstrated that the standard test method is not physiologically relevant by showing a lack of correlation between CoF measurements and cell damage on the urethral model postcatheterization.^20^

High friction between a catheter and the tissues it comes into contact with, upon catheterization, is common with the use of intermittent catheters, causing pain and discomfort for the patient.^7^ Materials with lower friction properties have been shown to reduce the incidence of trauma and damage associated with intermittent catheterization (4, 6, and 7). Coating the catheter with a hydrophilic coating is a widely used technique to reduce friction between the catheter and tissue upon catheterization.^3^ Previous studies have reported significantly lower frictional forces with the use of hydrophilic-coated urinary catheters in comparison to uncoated catheters.^4^ As expected, and in line with previous studies, the hydrophilic PVP-coated and hydrophilic IAS catheters demonstrated significantly lower CoF values than those of the uncoated PVC catheter. Significantly, no difference in CoF was found on comparison of the hydrophilic PVP-coated catheter with the IAS catheter, suggesting similar lubricious properties.

After catheterization of the urethral model at 0 min (catheter insertion), the percentage of cells remaining on the urethral model postcatheterization with the IAS catheter and the hydrophilic PVP-coated catheters (Brand 1, Brand 2, and Brand 4) was similar to the uncatheterized control indicating mild damage to the cell monolayer. However, after 2 min, mimicking in vivo indwell time, cell damage after catheterization with all the commercial catheters significantly increased due to catheter surface dry-out leading to increased tissue adhesion and/or friction during removal. Despite this, the IAS catheter caused significantly less damage to the cells in the urethral model than the uncoated and hydrophilic PVP-coated catheters (Brand 1, Brand 2, and Brand 3 [microeyelets] catheters).

This adhesive characteristic associated with PVP-coatings has been widely reported both in vitro and in vivo.^13,17^ A recent study compared the potential adhesion of intermittent catheter surfaces on catheter withdrawal. A range of commercial intermittent catheters, including a hydrophilic PVP-coated catheter and an IAS catheter, were investigated using an in vitro agar model. Catheters were hydrated, lowered into agar at a constant rate (5 mm s^–1^) and withdrawn after 2 min. Six out of eight hydrophilic PVP-coated catheters tested were observed to require a greater force to initiate withdrawal from the agar when compared to the IAS catheter, suggesting a relationship between PVP and adhesion.^17^ Moreover, in a randomized trial, 61 men tested four different hydrophilic PVP-coated catheters using them at random over 1 week. The severity of “sticking” experienced on catheter withdrawal was recorded using a three-point scale (not at all, a little, a lot). All four different hydrophilic PVP-coated catheters were reported to “stick” with comments from the subjects including “dried out fast and gripped on tight to the penis wall”.^13^ It is therefore, unsurprising that PVP has been employed in glues and adhesives.^26^

These findings first emphasize a relationship between catheter withdrawal and urethral microtrauma. This is a serious issue for patients with poor dexterity and spinal cord injury.^4^ Such users, who may need up to 15 min to catheterize, can find they have insufficient time to use PVP-coated catheters before they dry out.^2,18^ Second, the findings suggest that the use of IAS catheters instead of uncoated and hydrophilic PVP-coated catheters may help reduce urethral microtrauma experienced during catheter withdrawal from the bladder. The grading of damage to the cell monolayers after catheterization by each catheter further supports this suggestion (Table 3). Although all catheters were reported to exert cell damage to a degree, the IAS catheter was reported to exert slight to mild damage, whereas all of the hydrophilic PVP-coated catheters were graded mild to moderate.

Although the hydrophilic PVP-coated catheters removed cells from the monolayer, the binding of the cells to the coating was not strong. Moreover, the coatings along with cells may be sloughed off. Hydrophilic coatings have been reported to delaminate from the catheter surface due to shear force during the catheterization process.^27^ Previous work by Pollard et al. (2022) suggested that hydrophilic PVP-coatings can delaminate from intermittent catheters. Catheters were stained with red dye before insertion into an agar model for 2 min. After withdrawal, red dye remained within the agar suggesting shedding of the PVP-coatings from the catheter substrates.^17^

A similar general trend in cell adhesion was also observed. After 2 min contact (catheter withdrawal), all catheters showed an increase in percentage cell coverage on the surface. The surface of the catheters may have dried out enough for the coating to become sticky, causing adhesion between the catheter surface and the cell monolayer. Moreover, water may have evaporated from the surface of the IAS catheter, reducing the catheter’s lubricity. Nonetheless, cell adherence was evident to some extent on each catheter surface. There is limited assessment linking the relationship between the frictional characteristics of catheter surfaces and urethral microtrauma. Biering-Sørensen et al. (2001) attempted in a similar study to investigate urethral cell adherence to the surface of two commercial hydrophilic catheters, suggesting this was indicative of urethral trauma. After catheterization, catheters were stained, and cells on the surface were enumerated. However, similar to our study, no significant difference between the numbers of urethral epithelial cells on the surface of each catheter was found.^28^

Further observations in the urethral model indicated shedding of the hydrophilic PVP-coated catheter coatings, whereas delamination of the IAS catheter was not noted. Postcatheterization, PVP-coating was observed to remain behind on the urethral model, suggesting that hydrophilic PVP-coated catheters have the potential to leave coating residue behind in the urethra on withdrawal from a patient’s bladder. Importantly, the urethral model was catheterized once, however, in vivo intermittent catheterization is performed multiple times a day.^29,30^ This raises the question of whether delamination of hydrophilic PVP-coated catheters occurs in vivo and what clinical consequences accumulation of these residues might have. The clinical relevance and long-term significance of hydrophilic PVP-coated catheters’ adhesive characteristics, coating delamination and coating residues remain unknown.^13^ In 2019, the U.S. Food and Drug Administration (FDA) issued guidance on reporting delamination for intravascular catheter coatings.^31^ As a result of coating delamination, serious adverse events including pulmonary embolism, tissue necrosis, and death were reported. Current FDA analysis advocates premarket testing and device selection.^31^ Arguably, this provides a scientific premise for further studies specific to the relationship between hydrophilic coating delamination and the urethra. The fact that the IAS catheter did not delaminate in the urethral model suggests that these catheters may have an advantage over hydrophilic PVP-coated catheters in terms of potential issues surrounding coating delamination.

Limitations of the model used in our study are acknowledged. First, two fixed time points were conducted to mimic catheter insertion (0 min) and withdrawal of the catheter after in vivo indwell time (2 min). On average, the time to perform intermittent catheterization is less than 2–3 min.^32^ However, the process of real-life drainage of urine from the urethra via intermittent catheterization has been reported to take up to 5 min.^33^ Moreover, Leroux et al. (2021) investigated the time required to perform clean intermittent self-catheterization and reported durations ranging from 47 s to 11 min, 50 s.^32^ In our proposed model, 2 min may be insufficient, or alternatively too long and not fully representative of the complete process for all IC users. Second, the countersurface used in the model is a cell monolayer. This serves as a valid representation of the top epithelium barrier but is not fully representative of the multilayered human urethral mucosa.^34^ Furthermore, due to experimental constraints only one longitudinal section of the catheter was in contact with the cell monolayer countersurface, whereas within a patient the plane of the catheter shaft would be 360° surrounded by the urethra tissue. Future development could include incorporation of multilayered in vitro tissue which may be more clinically relevant and give further clinical insights. However, as Kazmierska et al. demonstrated in their model, a difference in frictional results for each intermittent catheter was observed between porcine aorta and porcine urethra tissue.^35^ This denotes the importance of choosing a clinically relevant countersurface but also indicates challenges from introducing additional variability and complexity. Nevertheless, it is still difficult to account for differences in urethral canal size, body temperature, moisture content, and the repeated frictional forces that occur during catheter insertion and withdrawal when testing in vitro.^18^ Furthermore, future studies will also investigate the performance of the in vitro model with intermittent catheters under nonideal conditions such as may be encountered during bacterial contamination of the urethral opening or during reuse of a catheter where coating damage and/or bacterial contamination could affect the frictional interface encountered between the urethra and catheter surface.

## 5 Conclusions

The CoF assay is the standard lubricity test used to assess lubricity of intermittent catheters; however, it fails to represent moist urethral conditions or account for urethral microtrauma. The in vitro biomimetic model presented allows for a more physiologically relevant comparison of intermittent catheters in terms of surface lubricity and the effect on urethral microtrauma. The majority of hydrophilic PVP-coated catheters caused significantly greater removal of cells from the urethral model after 2 min indwell time, compared to the IAS catheter, indicative of surface dry-out and catheter sticking. Moreover, hydrophilic PVP-coated catheters were shown to cause more cell damage than the coating-free IAS catheter. Interestingly, PVP-coating was shown to delaminate from the majority of the hydrophilic PVP-coated catheters and remain behind in the urethral model. The in vitro investigations indicated that the use of IAS catheters over hydrophilic PVP-coated catheters could reduce complications associated with intermittent catheterization, specifically, reduction of adhesive properties, and prevention of coating delamination and coating residues. Further investigations are necessary to fully understand the clinical impact of residual coatings and adhesive trauma on urethral tissue, including their potential role in the development of catheter-associated urinary tract infections.

## Data Availability

All data for this study are publicly available at: https://pure.qub.ac.uk/en/datasets/datasets-for-comparing-an-integrated-amphiphilic-surfactant-to-tr.

## Supporting Information Available

Representative images of T24 cell damage postcatheterization for all tested catheters at 0 min (insertion) and 2 min (withdrawal) (Figure S1) to support data contained in Table 3 (PDF)
