# Supplementary material for: Comparing an Integrated Amphiphilic Surfactant to Traditional Hydrophilic Coatings for the Reduction of Catheter-Associated Urethral Microtrauma
Source: ACS Omega. 2024 May 9;9(20):22410–22. doi: 10.1021/acsomega.4c02109 (PMC11112709; doi:10.1021/acsomega.4c02109)
Supplement: Supplementary file 1 — ao4c02109_si_001.pdf [file ao4c02109_si_001.pdf]

Supporting information for "Comparing an integrated amphiphilic surfactant to traditional hydrophilic coatings for the reduction of catheter-associated urethral microtrauma"

Jane Burns[1], David Pollard[2], Ased Ali [2], Colin P. McCoy[1], Louise Carson[1], Matthew P. Wylie[1]**

[1]*School of Pharmacy, Queen's University Belfast, 97 Lisburn Road, Belfast BT9 7BL, UK*

[2]*Convatec Limited, Convatec Technology Centre, First Avenue, Deeside Industrial Park, Deeside, Flintshire CH5 2NU, UK*

Appendix 1. Blinded visual scoring of cell damage after catheterization

A

i

ii

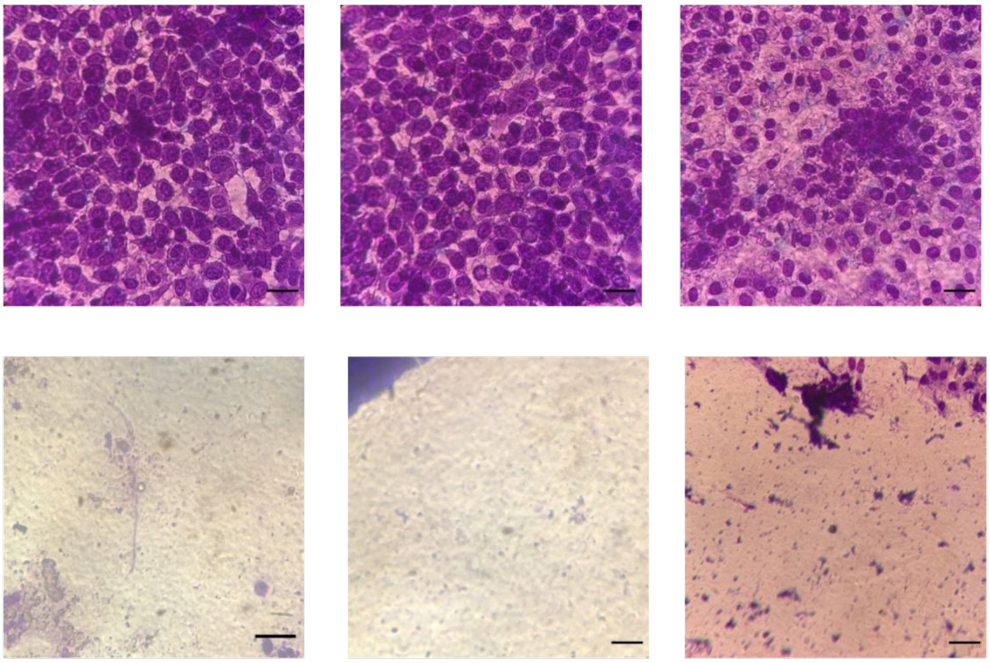

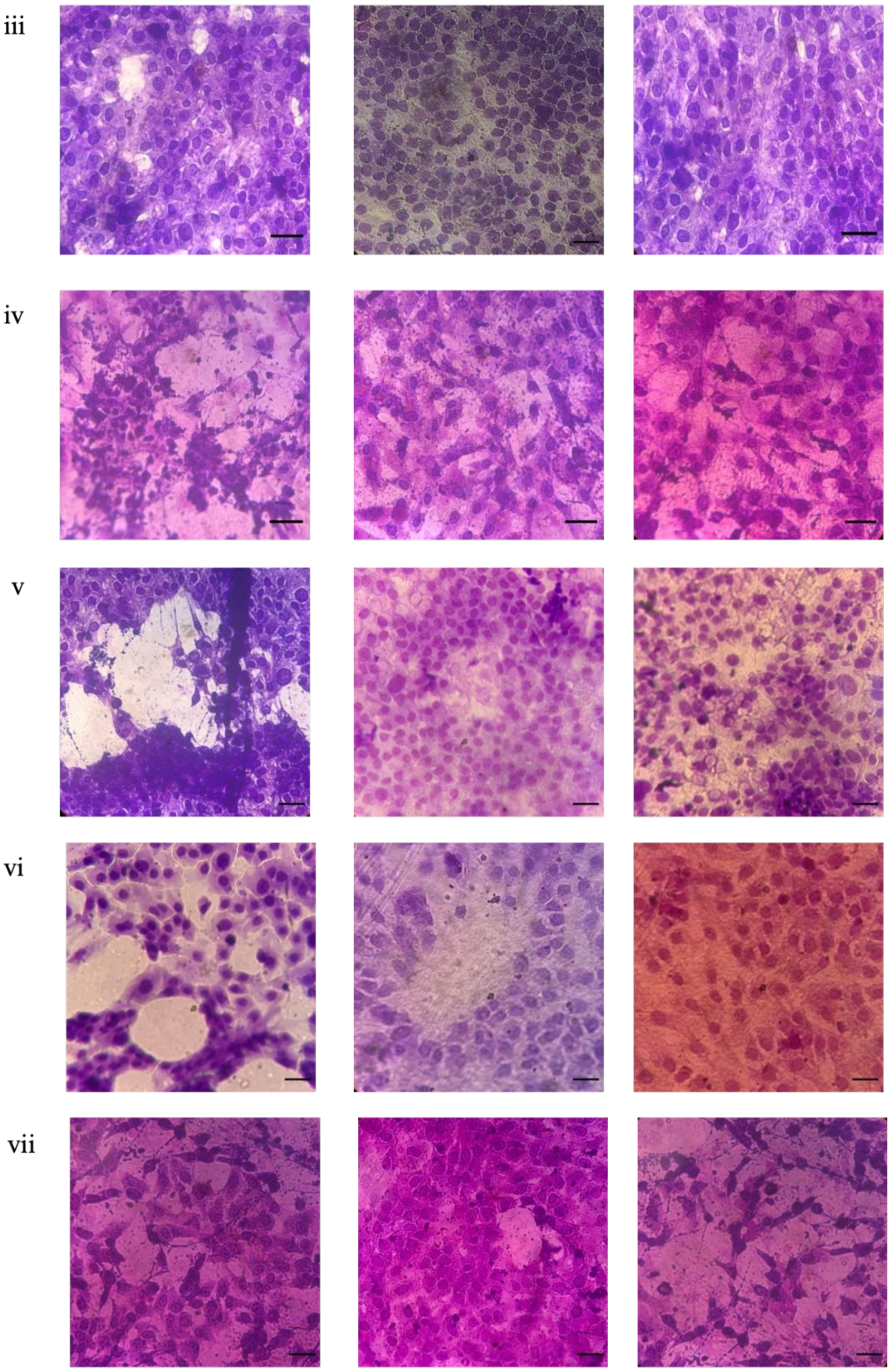
iii
iv
v
vi
vii

B

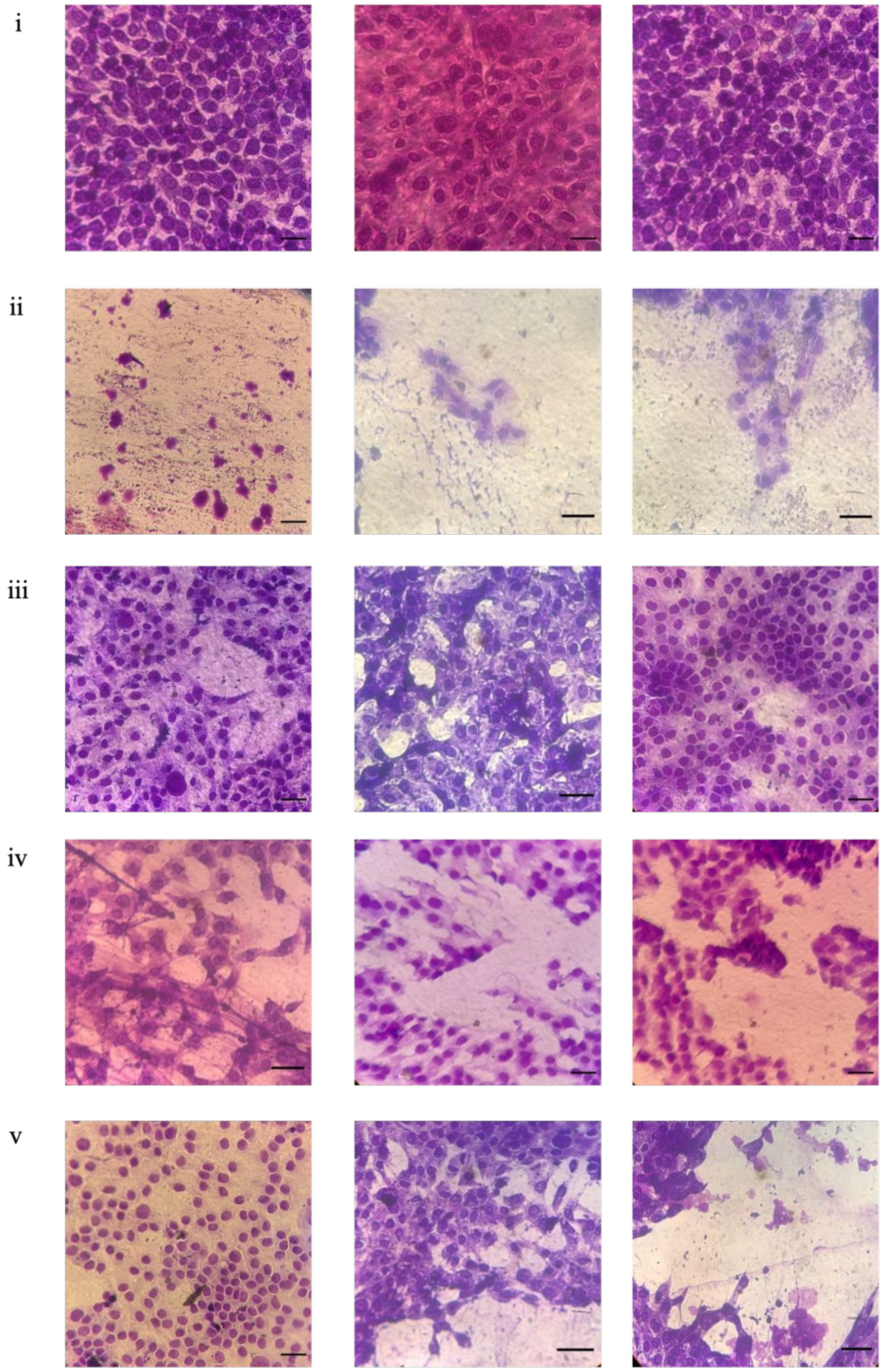

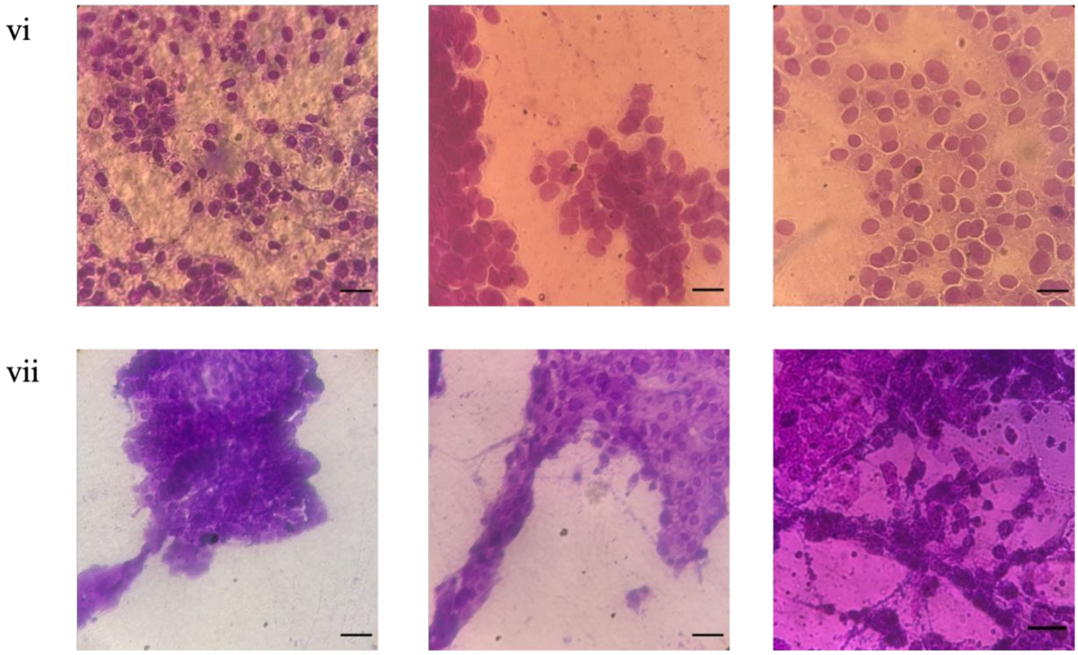

Figure S1: *T24 cell damage post catheterisation: cells remaining on the urethral model post-catheterisation. Catheters were placed onto the cell countersurface and advanced at 0 minutes (A), to mimic catheter insertion, or placed onto the cell countersurface for 2 minutes (B) and advanced, to mimic catheter withdrawal. Cells were stained with 0.1 % v/v crystal violet solution. (i) Uncatheterised control, (ii) uncoated, (iii) IAS catheter, (iv) Brand 1, (v) Brand 2, (vi) Brand 3 (microeyelets) and, (vii) Brand 4. Images acquired at x40 magnification. Scale bar 10 µm. Three representative images from twelve replicates shown.*
